# Supplementary material for: From sequence to enzyme mechanism using multi-label machine learning
Source: BMC Bioinformatics. 2014 May 19;15:150. doi: 10.1186/1471-2105-15-150 (PMC4229970; doi:10.1186/1471-2105-15-150)
Supplement: Additional file 2 — Java code of ml2db. Additional file ml2db_code.tar.gz contains the Java source code to run the multi-label machine learning experiments and save the results to database. The code’s Javadoc is included. [file 1471-2105-15-150-S2.zip › additional file 2/ml2db/utils/doc/uk/ac/standrews/utils/main/setutils/Set.html]

Set


JavaScript is disabled on your browser.


- Overview
- Package
- Class
- Use
- Tree
- Deprecated
- Index
- Help

- Prev Class
- Next Class

- Frames
- No Frames

- All Classes

- Summary:
- Nested |
- Field |
- Constr |
- Method

- Detail:
- Field |
- Constr |
- Method


uk.ac.standrews.utils.main.setutils

## Class Set

- java.lang.Object
- - uk.ac.standrews.utils.main.setutils.Set

- All Implemented Interfaces:
  :   java.lang.Comparable<Set>

  ---

    

  ```
  public class Set
  extends java.lang.Object
  implements java.lang.Comparable<Set>
  ```

  A set and its subsets. A set is a subset of a given set if all its elements
  belong to the set. For example: a-b, or a-c, or b-d are subsets of a-b-c-d,
  but a-e is not.

  Version:
  :   16 Mar 2011

  Author:
  :   Luna De Ferrari luna.deferrari-at-ed.ac.uk

- - ### Constructor Summary

    Constructors

    Constructor and Description

    `Set(java.lang.String id,
    java.util.TreeSet<java.lang.String> elements,
    SupersetsManager manager)`

    `Set(java.lang.String id,
    java.util.TreeSet<java.lang.String> elements,
    SupersetsManager manager,
    java.util.TreeSet<Set> subsets)`
  - ### Method Summary

    Methods

    | Modifier and Type | Method and Description |
    | --- | --- |
    | `void` | `addSubset(Set newsetToAdd)` |
    | `Set` | `clone()` |
    | `int` | `compareTo(Set otherSet)` |
    | `boolean` | `contains(Set set)` |
    | `java.lang.String` | `elementsToString()` |
    | `java.util.TreeSet<java.lang.String>` | `getElements()` |
    | `int` | `getElementsSize()` |
    | `java.lang.String` | `getId()` |
    | `java.util.TreeSet<Set>` | `getSubsets()` |
    | `int` | `getSubsetsSize()` |
    | `java.lang.String` | `toReadableString()` |
    | `java.lang.String` | `toString()` |

    - ### Methods inherited from class java.lang.Object

      `equals, getClass, hashCode, notify, notifyAll, wait, wait, wait`

- - ### Constructor Detail


    - #### Set

      ```
      public Set(java.lang.String id,
         java.util.TreeSet<java.lang.String> elements,
         SupersetsManager manager)
      ```


    - #### Set

      ```
      public Set(java.lang.String id,
         java.util.TreeSet<java.lang.String> elements,
         SupersetsManager manager,
         java.util.TreeSet<Set> subsets)
      ```
  - ### Method Detail


    - #### addSubset

      ```
      public void addSubset(Set newsetToAdd)
      ```


    - #### clone

      ```
      public Set clone()
      ```

      **Overrides:**
      :   `clone` in class `java.lang.Object`


    - #### compareTo

      ```
      public int compareTo(Set otherSet)
      ```

      **Specified by:**
      :   `compareTo` in interface `java.lang.Comparable<Set>`


    - #### contains

      ```
      public boolean contains(Set set)
      ```


    - #### elementsToString

      ```
      public java.lang.String elementsToString()
      ```


    - #### getElements

      ```
      public java.util.TreeSet<java.lang.String> getElements()
      ```


    - #### getElementsSize

      ```
      public int getElementsSize()
      ```


    - #### getId

      ```
      public java.lang.String getId()
      ```


    - #### getSubsets

      ```
      public java.util.TreeSet<Set> getSubsets()
      ```


    - #### getSubsetsSize

      ```
      public int getSubsetsSize()
      ```


    - #### toReadableString

      ```
      public java.lang.String toReadableString()
      ```


    - #### toString

      ```
      public java.lang.String toString()
      ```

      **Overrides:**
      :   `toString` in class `java.lang.Object`


- Overview
- Package
- Class
- Use
- Tree
- Deprecated
- Index
- Help

- Prev Class
- Next Class

- Frames
- No Frames

- All Classes

- Summary:
- Nested |
- Field |
- Constr |
- Method

- Detail:
- Field |
- Constr |
- Method
